# Collaborative safety: The impact of patient and caregiver engagement in perioperative care

**DOI:** 10.1016/j.pecinn.2026.100494

**Published:** 2026-07-16

**Authors:** Caoimhe C. Duffy, Justin B. Ziemba, Pavel Karasek, Gary A. Bass

**Affiliations:** a Department of Anesthesiology & Critical Care, Perelman School of Medicine at University of Pennsylvania, Philadelphia, PA, USA; b Leonard Davis Institute for Health Economics, University of Pennsylvania, Philadelphia, PA, USA; c Center for Perioperative Outcomes Research and Transformation (C-PORT), University of Pennsylvania, Philadelphia, PA, USA; d Department of Clinical Effectiveness and Quality Improvement, Hospital of the University of Pennsylvania, Philadelphia, PA, USA; e Division of Urology, Department of Surgery, Hospital of the University of Pennsylvania, Perelman School of Medicine, University of Pennsylvania, Philadelphia, PA, USA; f St. Olaf College, Northfield, MN, USA; g Division of Traumatology, Surgical Critical Care and Emergency Surgery, Perelman School of Medicine, University of Pennsylvania, Philadelphia, PA, USA; h Department of Biostatistics, Epidemiology and Informatics, Perelman School of Medicine, University of Pennsylvania, Philadelphia, PA, USA

**Keywords:** Patient engagement, Perioperative safety, Clinical decision-making, Resilience-based intervention, Patient activation

## Abstract

**Objective:**

Patient involvement in perioperative safety is increasingly emphasized, yet opportunities for patients and caregivers to participate meaningfully are often constrained by clinical workflows and limited role clarity. We adapted the One Safe Act (OSA) approach, a resilience-based intervention originally designed for staff, to evaluate whether a simple reflective prompt could foster patient and caregiver engagement.

**Methods:**

We conducted an exploratory quality-improvement study in the Perioperative Care Unit between February and August 2024. After routine surgical and anesthesia review, patients and caregivers were invited to identify one action they used or planned to use to promote safety and to complete brief survey items (awareness of safety, perceived role, comfort asking questions, and trust in facility efforts). Free-text responses underwent inductive thematic analysis. Associations between attitudes and behaviors were examined using Fisher's exact test with false-discovery-rate adjustment. Proactive engagement was also assessed at the respondent level.

**Results:**

We collected 122 OSA responses (98 patients, 24 caregivers). Most respondents reported awareness of patient safety (71%), endorsed a personal role (90%), felt comfortable asking questions (94%), and trusted the facility's safety efforts (84%). A composite high-engagement profile (“Proactivity Trust”) was met by 28%. Eight behavioral themes emerged, most commonly dietary adjustments (33%), strategic planning (32%), and medication adherence (28%); 3% of responses described no specific action. When classified by engagement type, 58% of coded behaviors were routine and 41.8% were proactive. At the respondent level, 40.8% of patients and 45.8% of caregivers reported at least one proactive behavior (*p* = 0.65). Strong belief in a personal role was associated with greater comfort asking questions (*p* = 0.001), and “Proactivity Trust” was associated with greater safety knowledge (*p* = 0.002).

**Conclusions:**

Although most patients recognized a role in safety, fewer reported proactive engagement. The OSA prompt elicited patient- and caregiver-generated safety behaviors and identified opportunities to strengthen engagement.

**Practice implications:**

Embedding OSA in preoperative counseling may provide a scalable approach to normalize patient- and caregiver-initiated safety behaviors and strengthen communication at a vulnerable point in care.

## Highlights

• Patients and caregivers are active partners in perioperative safety, not passive recipients of care.
• A brief “One Safe Act” (OSA) prompt asks them to name a specific action they will take to protect themselves
• Common OSAs include adhering to fasting instructions, bringing updated medication lists and planning safe transport home.
• The OSA tool is low-cost and scalable, catalyzing reflection, shared decision-making, and teach-back during preoperative counseling.
• OSA responses surface patient priorities and gaps, enabling clinicians to tailor education and close safety risks.

## Glossary

PCU: Perioperative Care Unit
OSA: One Safe Act
SRQR: Standards for Reporting Qualitative Research

## 1 Introduction

Patients, advocacy groups, healthcare institutions, and policymakers increasingly emphasize meaningful patient and caregiver involvement in treatment decision-making as a cornerstone of high-quality care [1], [2], [3], [4]. When patients actively participate, by asking questions, expressing concerns and engaging in choices, they experience fewer errors and adverse events and gain a greater sense of agency in the often overwhelming hospital environment [5], [6], [7], [8]. These advantages are particularly evident during the perioperative phase of care, where patients transition through multiple teams and handoffs within a high-risk pathway [9], [10]. Many patients hesitate to speak up about their care, trusting that the healthcare system will act in their best interests [6], [11], [12]. This deference overlooks the inevitability of human error and the need for transparent, collaborative safety partnerships [9], [13], [14], [15], [16], [17], [18]. Despite broad endorsement, translating patient and caregiver engagement into routine practice remains challenging [1], [12], [15], [19].

Existing approaches to patient engagement in safety are often clinician-directed relying on instructions, checklists, or prompts that ask patients to comply with predefined safety tasks [20]. Yet patients and caregivers may already engage in self-defined behaviors, small, intuitive actions they believe will help keep care safe, and these behaviors are rarely elicited or described in perioperative settings. Making these patient-generated practices visible is essential for designing realistic, personalized interventions that foster effective engagement.

The One Safe Act (OSA) intervention is a proactive resilience-based intervention originally developed for perioperative staff to identify and share concrete safety behaviors used in everyday work [21], [22]. In this study, we adapted OSA framework into a patient-facing reflective prompt, inviting patients and caregivers to identify one action they used or planned to use to support safety during the perioperative period. We aimed to examine what patients and caregivers understand as their role in perioperative safety, what safety-related behaviors they identify when prompted to name a one safe act and whether reported attitudes toward safety are associated with proactive forms of patient engagement.

## 2 Methods

We conducted an exploratory mixed-methods quality-improvement study to examine responses to a patient-facing adaptation of the OSA prompt in the preoperative setting. The study is grounded in experiential learning theory, which suggests that patient engagement in safety behaviors is facilitated through the acquisition of knowledge and the process of self-reflection [23] situated within the context of the clinical perioperative environment. By encouraging patients to reflect on their own actions, the intervention fosters a deeper understanding of their role in maintaining safety.

### 2.1 Setting, context and study population

This study was conducted within the perioperative care unit (PCU) of the Hospital of the University of Pennsylvania over 7 months (February–August 2024). All patients scheduled for an elective surgical or interventional procedure in the PCU were eligible for inclusion. However, to ensure uniformity in patient prompting, recruitment only occurred when an activity facilitator (authors CCD and JBZ) was present on the unit. Therefore, a convenience sample of patients were selected for participation from among those electively scheduled for surgical or interventional procedure. No patient participated more than once. The activity was conducted exclusively prior to a surgical or interventional procedure. Either a patient or their present caregiver could participate. Institutional Review Board approval was attained by the University of Pennsylvania with a waiver for informed consent.

### 2.2 Activity design

**Fig. 1:**
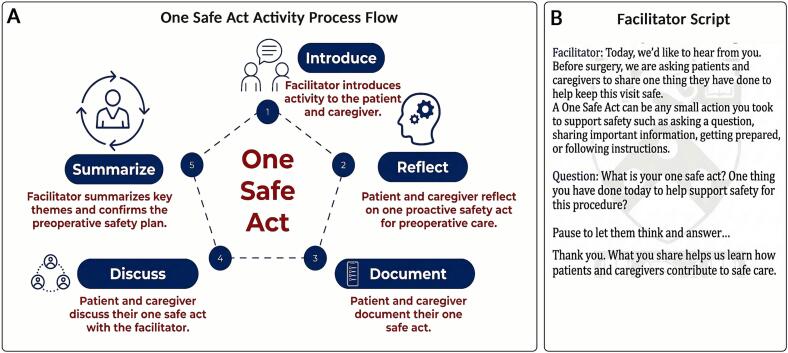
Outline of One Safe Act activity process flow and facilitator script.

The intervention was adapted from the previously described OSA intervention for perioperative staff [21], [22]. Fig. 1 outlines the activity and provides a facilitator script. During routine preoperative assessment in the PCU, a local clinical leader (CCD or JBZ) invited patients (and, when present, caregivers) to participate in OSA after the standard surgical and anesthesia review. With verbal permission to proceed, the facilitator introduced the OSA activity using a brief script, defining an OSA as a concrete action or behavior used to promote one's own safety. The facilitator modeled the task by sharing a personal OSA from clinical practice, then paused to allow reflection. Participants were not provided with a narrow operational definition of safety. Instead, they were invited to identify any action they believed supported their safety during the perioperative period, allowing us to examine how patients and caregivers themselves construed safety in this context. Participants were asked to identify their own OSA, defined as an action they had taken or intended to take to support safety during the perioperative period.

### 2.3 Data collection

Responses were recorded by participants on their mobile devices using a secure, web-based REDCap (Research Electronic Data Capture) survey hosted by the University of Pennsylvania, immediately following the OSA prompt. The survey collected respondent role (patient or caregiver), a free-text response describing the participant's OSA, and several structured items related to patient safety. These included whether the participant had heard of patient safety, whether they believed they had a role in patient safety, how comfortable they felt asking questions when something did not seem right, and whether they agreed that the facility actively looks for ways to improve patient safety. The survey also captured the type of healthcare service received that day and the care location. A copy of the survey instrument is provided in Appendix A. All responses were captured anonymously. To protect participant privacy and encourage participation no demographic data was collected.

### 2.4 Quantitative data analysis

Structured survey responses were summarized descriptively. Ordinal response items, including comfort asking questions when something did not seem right, were collapsed into binary variables where appropriate. Associations between categorical variables were tested using Fisher's exact test. To account for multiple comparisons, the Benjamini–Hochberg procedure was applied with a false-discovery-rate threshold of 5% [24]. A composite binary variable, termed “Proactivity Trust,” was defined as positive only when respondents strongly endorsed three criteria: belief in a personal role in safety, comfort in asking questions, and trust that the facility actively improves patient safety. Because participants could report multiple safety behaviors, analyses were conducted at two levels. The primary inferential analysis used the respondent as the unit of analysis, classifying each participant according to whether they reported at least one proactive behavior. A secondary descriptive analysis was performed at the level of coded behaviors to characterize the distribution of routine and proactive actions. Comparisons between patients and caregivers were performed using Fisher's exact test. Behavior-level analyses were not subjected to formal statistical testing because observations were not independent. All analyses were limited to bivariate associations; no multivariable modeling was performed. Statistical analyses were conducted in R version 4.4.1 (R Foundation for Statistical Computing), using the tidyverse package for data handling [25].

### 2.5 Qualitative data analysis

Free-text responses describing participants' OSA were analyzed thematically. Responses were first reviewed to determine whether they contained a single behavior or multiple distinct behaviors. When multiple behaviors were described, each was separated for individual coding. Preliminary codes were developed through consensus by PK, JBZ, and CCD and then independently applied across the dataset by all three authors. Up to five codes could initially be assigned to a single response to capture the breadth of behaviors described. The coding framework is summarized in Table 1.

**Table 1:** Categorical analysis of One Safe Acts performed by patients, with examples. This table summarizes the eight inductively derived categories used to code preoperative One Safe Act (OSA) narratives from patients and caregivers, with brief operational definitions and representative verbatim examples for each category. Where relevant, examples are labeled as proactive (“extra engagement”) when they exceeded routine, procedure-required preparation, or as routine compliance when they aligned with standard preoperative instructions. Some narratives included more than one behavior; in such cases, actions were disaggregated and coded separately, so category totals may exceed 100% of responses. OSA denotes One Safe Act.

Category	Description	Example
Dietary adjustments	Adjusting diet. Fasting, adjusting drinking regime, abstaining from alcohol.	*“Fasting and getting here on time”* *“Increased nutrition, added protein”*
Strategical preparation/planning	Logistical adjustments, like making house more accessible or preparing meals, transportation arrangements, bringing helpful items, paperwork, and other kinds of practical planning or strategical behavior.	*“Clear path to bed”*
Medication adjustments/adherence	Stopping, starting, adjusting, or monitoring medication usage.	*“Checking to make sure all meds are taken correctly.”*
Religious/meditation/mental preparation	Prayer, meditation, precontemplation, self-talk, breath-work, supporting loved one, other social or physical activity intended to relax the respondent.	*“Mostly mental preparedness as I am also in the midst of a cancer battle. Meditation.”* *“Praying for comfort. Safety and protection”*
Following instructions	Following instructions or reading mandatory instructions directly mentioned.	*“Followed all pre op instructions”* *“Fasting and getting here on time”*
Education/advocacy	Asking questions, preparing questions, speaking up, education extra to reading basic instructions.	*“I asked questions of my healthcare team before the surgery.”*
Hygiene	Showering, washing hands, masking, removing piercings or shaving	*“Shave in surgical area”* *“Showered”*
Physical adjustments/preparation	Working out, walking, rest, other activity intended to get patient into better physical condition	*“Increased mobility, strength exercises.”*
Nothing	Empty response or patient indicated doing nothing	*“I did nothing out of the ordinary.”*

The research team then met to compare coding decisions and review primary and, where relevant, secondary and tertiary codes. Codes were refined iteratively through discussion and consensus using an inductive approach grounded in participants' own descriptions. This process was repeated over three additional rounds until stable themes were identified. In addition to thematic coding, each response was dichotomized to identify proactive engagement, defined as a response describing an active, self-initiated behavior to support safety, such as asking questions, seeking clarification, or obtaining additional information. This was distinguished from responses describing more routine, preparatory, or logistical actions related to the healthcare visit which appeared more frequently in the dataset.

## 3 Results

A total of 122 One Safe Act (OSA) surveys were completed, including 98 by patients and 24 by caregivers in the PCU. Although each participants submitted a OSA free-text response, some responses described multiple actions.

### 3.1 Survey responses

Seventy-one percent of participants reported awareness of patient safety, 21% indicated no awareness, and 8% were unsure. Ninety percent affirmed that they had a role in ensuring their safety, and 94% reported being comfortable asking questions most of the time or always. Eighty-four percent expressed trust that the facility worked to improve patient safety, whereas 9% strongly disagreed. Twenty-eight percent met criteria for “Proactivity Trust,” defined as strongly endorsing a personal role, reporting comfort in asking questions, and expressing trust in institutional safety efforts.

### 3.2 Qualitative responses

**Fig. 2:**
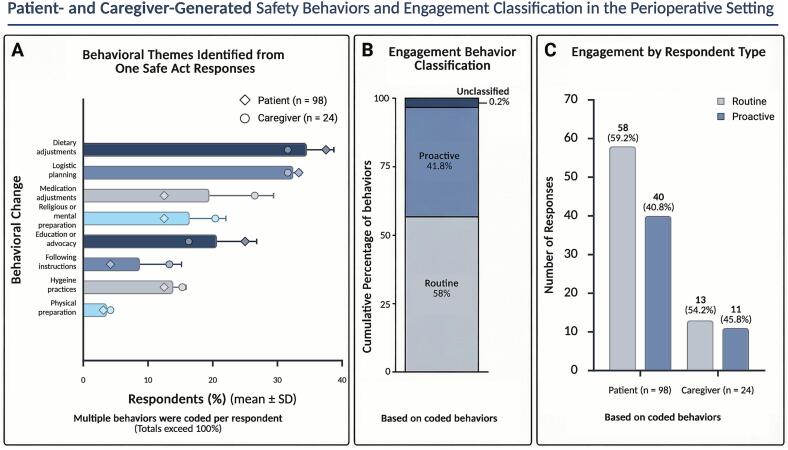
Patient- and caregiver-generated safety behaviors and engagement classification in the perioperative setting. Panel A shows thematic categories from 122 One Safe Act (OSA) responses (98 patients, 24 caregivers); responses could include multiple behaviors, and percentages exceed 100%. Panel B shows the proportion of behaviors classified as routine, proactive, or no action. Panel C compares routine and proactive behaviors between patients and caregivers; inferential comparisons are based on respondent-level analysis. Percentages in Panels B and C are based on coded behaviors. OSA = One Safe Act.

Thematic analysis identified eight categories of patient- and caregiver-reported safety behaviors (Table 1; Fig. 2A). Dietary adjustments (33%) and logistical or strategic planning (32%) were most frequent, followed by medication adherence (28%). Less common categories included religious or mental preparation (19%), education or advocacy (16%), explicit adherence to instructions (16%), hygiene practices (13%), and physical preparation (12%). A small proportion of responses (3%) described no specific action. When classified by engagement type, 58% of coded behaviors were routine, defined as preparatory or procedure-related actions, whereas 41.8% were proactive, defined as self-initiated behaviors such as question-asking, information-seeking, or advocacy (Fig. 2B). These estimates are based on coded behaviors and are descriptive.

For inferential analysis, the respondent was used as the unit of analysis. Among patients (*n* = 98), 40 (40.8%) reported at least one proactive behavior, compared with 11 of 24 caregivers (45.8%); this difference was not statistically significant (Fisher's exact test, *p* = 0.65). Behavior-level comparisons were directionally consistent with these findings (Fig. 2C). Among patient-reported behaviors, 59.2% were routine and 40.8% proactive, whereas among caregiver-reported behaviors, 54.2% were routine and 45.8% proactive. Because multiple behaviors could arise from a single respondent, these estimates are not independent and are presented for descriptive comparison only. The proportion of proactive behaviors was modestly higher among caregivers than patients, but the magnitude of this difference was small and did not reach statistical significance. Strong belief in a personal role in safety was associated with greater comfort in asking questions (*p* = 0.001), and participants meeting “Proactivity Trust” criteria were more likely to report knowledge of patient safety (*p* = 0.002). No other associations reached statistical significance.

## 4 Discussion

This study revealed a broad range of safeguarding behaviors that patients and caregivers described in the preoperative setting. When prompted to identify a concrete action that supported safety, most participants described behaviors aligned with procedural adherence and preparation, while a smaller proportion articulated more proactive forms of engagement such as question-asking, information-seeking, advocacy, or anticipatory planning. Importantly, participants were not given a narrow operational definition of safety; instead, they were invited to identify any action they believed supported their safety during the perioperative period. Their responses therefore provide insight into how patients and caregivers themselves construed safety across preparation, the immediate surgical encounter, and early recovery-related planning. Together, these findings suggest that patients often anchor their safety efforts in explicit clinician guidance, highlighting the importance of clear communication in fostering engagement at a time of heightened vulnerability [11], [26]. They are also consistent with prior work identifying risk elements considered important for inclusion in a patient-driven surgical safety checklist [27], [28].

A substantial minority described proactive behaviors, including preparing questions, seeking information, and engaging in mental, spiritual, or physical preparation. These responses suggest that patients often conceptualize safety more broadly than the prevention of technical error alone, encompassing readiness, communication, self-advocacy and personal preparation for surgery [5]. Prior work has shown that structured interventions encouraging question-asking improve patient confidence and the likelihood of voicing concerns [28], [29]. This suggests that strategies used in patient-centered communication and shared-decision making may also be relevant to safety from the patient's perspective [30]. In our study, however, only a subset of patients reached this level of active participation without prompting, suggesting a gap between endorsement of safety and enactment of proactive safety behaviors. This interpretation is consistent with prior literature describing barriers to engagement, including deference to authority and fear of disrupting workflow [30], [31], [32]. For educators and clinicians, this finding reinforces the need for communication strategies that both normalize patient input and recognize the broader ways patients themselves understand and support safety.

Caregivers emerged as important advocates, with nearly one quarter reporting educational or advocacy activities on behalf of patients. Caregivers demonstrated a modestly higher proportion of proactive behaviors than patients; however, this difference did not reach statistical significance and should be interpreted as hypothesis-generating. This aligns with evidence that family presence and advocacy can enhance communication and reduce perioperative anxiety [33], [34]. Unlike patients, caregivers may feel less constrained by hierarchical dynamics and more comfortable questioning providers. Harnessing this role by explicitly inviting caregiver participation offers a practical route to strengthen safety partnerships, particularly for patients hesitant to speak up [35].

Thematic categories such as meditation, prayer, exercise, and positive self-talk reflect psychosocial strategies that enhance resilience and self-efficacy attributes associated with greater willingness to raise concerns [36], [37], [38], [39]. Integrating encouragement of these practices into preoperative education may therefore represent an overlooked opportunity to build engagement. Similarly, participants who engaged in strategic logistical planning, such as arranging transportation or preparing the home environment, demonstrated a sense of control over their care trajectory, echoing prior work showing that patients perceiving themselves as active participants are more likely to adopt safety behaviors [40], [41]. The observed association between strong belief in a personal role and comfort asking questions further supports this linkage between self-efficacy and communication [42], [43].

Several limitations warrant caution. The study was conducted in a single academic center, limiting generalizability to other contexts. Responses were collected immediately prior to surgery, potentially biasing toward recent instructions rather than longer-term safety attitudes. Demographic and procedural data were intentionally not collected, preventing exploration of variation by age, prior experience, or surgery type. Recruitment was opportunistic and depended on facilitator availability, raising the possibility of selection bias. Finally, the absence of follow-up interviews constrained interpretation of the depth and meaning of responses. Despite these limitations, the study provides rare insight into the safety behaviors patients and caregivers report spontaneously in a high-risk environment.

### 4.1 Innovation

This study adds to the patient-safety literature by adapting a clinician-facing, resilience-based intervention into a patient- and caregiver-facing reflective prompt within perioperative care. Rather than prescribing predefined safety tasks, the OSA prompt elicits how participants themselves understand and support safety. In doing so, it makes visible a broader range of patient- and caregiver-generated behaviors, from routine adherence and practical preparation to advocacy, anticipatory planning, and psychosocial readiness, while also highlighting caregiver participation as an underrecognized resource for perioperative safety. Its contribution lies less in claiming novelty about patient engagement itself and more in demonstrating that a brief, low-burden reflective prompt can surface patient-generated safety behaviors at a vulnerable point in care. Because the question is simple and does not require specialized training or infrastructure, it may also represent a low-cost, scalable approach that could be incorporated into routine preoperative interactions by a range of team members.

### 4.2 Conclusion

Patients and caregivers in the preoperative setting described safety through a broad range of self-defined behaviors, most often related to preparation, adherence to guidance, and practical readiness, with a smaller proportion identifying more proactive forms of engagement such as question-asking, information seeking, and advocacy. Caregivers emerged as important safety partners, often assuming advocacy roles on behalf of patients. By adapting the One Safe Act (OSA) prompt for preoperative patients and caregivers, this study highlights a simple way to surface how participants themselves understand and support safety at a vulnerable point in care. These findings suggest that preoperative education and communication strategies should move beyond procedural instruction alone to more explicitly invite patient and caregiver participation as part of collaborative perioperative safety.

## CRediT authorship contribution statement

**Caoimhe C. Duffy:** Writing – review & editing, Writing – original draft, Supervision, Project administration, Methodology, Investigation, Data curation, Conceptualization. **Justin B. Ziemba:** Writing – review & editing, Writing – original draft, Supervision, Project administration, Methodology, Conceptualization. **Pavel Karasek:** Writing – review & editing, Writing – original draft, Formal analysis. **Gary A. Bass:** Writing – review & editing, Writing – original draft, Methodology, Formal analysis.

## Funding

This work was supported by the Leonard Davis Institute for Health Economics Summer Undergraduate Research Program (Philadelphia, PA), which provided stipend support and mentorship to PK. This work was supported in part by an APSF/FAER Mentored Research Training Grant awarded to Dr. Duffy in 2024.

## Declaration of competing interest

The authors declare that they have no known competing financial interests or personal relationships that could have appeared to influence the work reported in this paper.

## Data Availability

De-identified data and code are available from the corresponding author on reasonable request.

## Appendix A Supplementary data

Supplementary material: Example of survey template distributed to patients and their care givers.
