# Supplementary material for: Collaborative safety: The impact of patient and caregiver engagement in perioperative care
Source: PEC Innov. 2026 Jul 16;9:100494. doi: 10.1016/j.pecinn.2026.100494 (PMC13425881; doi:10.1016/j.pecinn.2026.100494)
Supplement: Supplementary file 1 — Supplementary material: Example of survey template distributed to patients and their care givers. [file mmc1.pdf]

One Safe Act

Date

____

Are you a patient or caregiver?

- ◯ Patient
- ◯ Caregiver
- ◯ Neither

Everyday, across healthcare, there are clinical and non-clinical staff who are collectively doing "the small things" for your safety. These ONE SAFE ACT demonstrate our commitment to doing what's right to safeguard your health and safety. This is called patient safety.

However, you are an equal partner in protecting your health and safety.

We want to hear from you about about your ONE SAFE ACT.

Everyday, across healthcare, there are clinical and non-clinical staff who are collectively doing "the small things" for your loved one's safety. These ONE SAFE ACT demonstrate our commitment to doing what's right to safeguard your loved one's health and safety. This is called patient safety.

However, as a caregiver you are an equal partner in protecting your love one's health and safety.

We want to hear from you about about your ONE SAFE ACT.

A ONE SAFE ACT can be any task that you performed to safeguard your health and safety leading up to or during your healthcare visit today.

What is your ONE SAFE ACT?

____
(Please write a short description.)

A ONE SAFE ACT can be any task that you performed to safeguard the health and safety of your loved one leading up to or during their healthcare visit today.

What is your ONE SAFE ACT?

____
(Please write a short description.)

Have you heard of patient safety?

- ◯ Yes
- ◯ No
- ◯ Don't know

Do you believe you have a role in patient safety?

- ◯ Yes
- ◯ No
- ◯ Don't know

Do you feel comfortable asking questions when something doesn't seem right.

- ◯ Never
- ◯ Rarely
- ◯ Sometimes
- ◯ Most of the time
- ◯ Always
- ◯ Don't know

This facility actively looks for ways to improve patient safety.

- ◯ Strongly disagree
- ◯ Disagree
- ◯ Neither agree or disagree
- ◯ Agree
- ◯ Strongly agree
- ◯ Don't know

What healthcare service did you have performed today?	◯ Hospital admission<br>◯ Medical office consultation<br>◯ Emergency department visit<br>◯ Surgical, invasive, or endoscopic procedure<br>◯ Laboratory visit<br>◯ Radiology test<br>◯ Other
What healthcare service did your loved one have performed today?	◯ Hospital admission<br>◯ Medical office consultation<br>◯ Emergency department visit<br>◯ Surgical, invasive, or endoscopic procedure<br>◯ Laboratory visit<br>◯ Radiology test<br>◯ Other
If other, please describe.	__________________________________
If other, please describe.	__________________________________
Please select the location of your healthcare services today.	◯ Hospital of the University of Pennsylvania (HUP) or Perelman Center for Advanced Medicine (PCAM)<br>◯ Presbyterian Medical Center (PPMC) or Penn Medicine University City (PMUC)<br>◯ Pennsylvania Hospital (PAH) or Penn Medicine Washington Square (PMWS)<br>◯ Other
Please select the location of your loved one's healthcare services today.	◯ Hospital of the University of Pennsylvania (HUP) or Perelman Center for Advanced Medicine (PCAM)<br>◯ Presbyterian Medical Center (PPMC) or Penn Medicine University City (PMUC)<br>◯ Pennsylvania Hospital (PAH) or Penn Medicine Washington Square (PMWS)<br>◯ Other
